# Kinematic analysis of the back squat at different load intensities in powerlifters and weightlifters

**DOI:** 10.3389/fspor.2024.1454309

**Published:** 2024-10-31

**Authors:** Valerio Giustino, Domenico Savio Salvatore Vicari, Flavia Figlioli, Marco Gervasi, Eneko Fernández Peña, Naima Schifaudo, Mattia Tedesco, Patrik Drid, Antonio Paoli, Giuseppe Battaglia, Antonino Bianco, Antonino Patti

**Affiliations:** ^1^ Sport and Exercise Sciences Research Unit, Department of Psychology, Educational Science and Human Movement, University of Palermo, Palermo, Italy; ^2^ Department of Neurosciences, Biomedicine and Movement Sciences, University of Verona, Verona, Italy; ^3^ PhD Program in Health Promotion and Cognitive Sciences, University of Palermo, Palermo, Italy; ^4^ Department of Biomolecular Sciences—Division of Exercise and Health Sciences, University of Urbino Carlo Bo, Urbino, Italy; ^5^ Department of Physical Education and Sport, University of the Basque Country UPV/EHU, Vitoria-Gasteiz, Spain; ^6^ A.S.D. Palermo Sport & Performance, Palermo, Italy; ^7^ Faculty of Sport and Physical Education, University of Novi Sad, Novi Sad, Serbia; ^8^ Department of Biomedical Sciences, University of Padua, Padua, Italy; ^9^ Regional Sports School of Italian National Olympic Committee (CONI) Sicilia, Palermo, Italy

**Keywords:** biomechanics, kinematics, back squat, powerlifting, weightlifting

## Abstract

**Introduction:**

This study aimed to evaluate the angular kinematics of the hip, knee, ankle, and the linear kinematics of the barbell during the back squat (BS) at different load intensities in powerlifters and weightlifters.

**Methods:**

Seventeen athletes were recruited (*n* = 14 powerlifters; *n* = 3 weightlifters). The 1-RM of the BS of each participant was calculated and, 1-week after, each participant was asked to perform 5 trials of the BS at different load intensities (i.e., 60%, 70%, 80%, 90%, 100%) of the 1-RM. An action camera recorded the execution of each BS trial in the sagittal plane and, afterward, the videos were analyzed by measuring the range of motion (ROM) of hip, knee, and ankle for the angular kinematics, and the timing, distances, speeds, and accelerations of the barbell for the linear kinematics.

**Results:**

Regarding the angular kinematics, no significant differences were found in the parameters in the starting and ending positions among the 5 trials, while a significant decrease was found in the hip relative angle (*p* = 0.026) in the maximum flexion position as load intensity increased. Regarding the linear kinematics, a significant difference was found in the descent acceleration (*p* = 0.049) in the descent phase, while a significant difference was found in the ascent speed (*p* < .001) and vertical speed of ascent (*p* < .001) in the ascent phase, which decreased as load intensity increased.

**Discussion:**

Our findings show that the angular and linear kinematics of BS change as load intensity increases.

## 1 Introduction

The squat is considered one of the best exercises to improve physical fitness as its execution requires the simultaneous recruitment and coordination of multiple muscle groups of the trunk and lower limbs (1). In fact, it is a multijoint exercise that mainly involves the hips, knees, and ankles, and which athletes of different sports perform to train the strength of lower limbs and back muscles (2–4). The squat can be a bodyweight exercise, or it can be performed with a barbell. Regarding the latter it is possible to distinguish the back squat and the front squat depending on the position of the barbell which can be positioned either across the back resting on the shoulders (back squat) or in front of the chest at the level of the clavicles (front squat) (5, 6).

Powerlifting and weightlifting are sports in which athletes during competitions are engaged in lifting the maximum weight possible. For each competition exercise, athletes have three attempts to lift the maximum weight possible in a single repetition. Powerlifting competition exercises are squat, bench press, and deadlift (7). Although in weightlifting the squat is not included among the competition exercises [which are instead the snatch and the clean and jerk (8)], it is included in the training programs as complementary exercise (9).

In the starting position of the squat, which coincides with the ending position, the athlete is in upright position with the knees and hips almost completely extended (3). The execution of the gesture should be a fluid movement that involves a descent phase, which ends when it is possible to draw a parallel line to the ground from the hip to the knee, and a subsequent ascent phase, which ends when it is possible to draw a perpendicular line to the ground from the hip to the knee (3). To be valid, the squat must reach a depth in which the top surface closest to the hip must drop below the top surface of the knee before starting the ascent phase. In fact, the optimal depth of the squat, i.e., the parallel squat or full squat, occurs when the descent phase ends with the thighs parallel to the ground or below this line (10, 11).

Given the widespread use of the squat among athletes, there are several studies that have analyzed the biomechanics of the gesture with the aim of improving performance and preventing injuries (1, 3, 12–18). In fact, it is recommended to perform the squat, which is a closed kinetic chain exercise, with minimal anterior knee displacement in order to optimize performance and reduce the risk of injury (19–22). Another recommendation is to maintain the tibia in an upright position because it would appear to reduce internal forces at the knee and emphasize recruitment of the hip extensor muscles (2). Zawadka et al. analyzed the relationship between the range of motion (ROM) of ankle, knee, hip, pelvis, and spine (measured in the sagittal plane) and timing parameters during a squat to its depth (23). The results showed that squat depth depends mainly on knee ROM. For this reason, the knee flexion angle could very well describe the depth of the squat. Furthermore, the authors report that pelvis and ankle timing can influence the maintenance of balance during the squat allowing the achievement of the adequate squat depth. Schoenfeld analyzed the effects of fatigue on squat kinetics and kinematics (16). In this way, a previous research suggested that exercises to exhaustion could lead to knee instability and thus increase the risk of injury (24). In line with the abovementioned findings, our previous study, aimed to analyze the postural control of athletes during the back squat (BS) at different intensities, showed that as the intensity increased the athletes had worse postural control (25). Thus, as is known, the intensity is a parameter that plays a fundamental role in the kinematics of the technical gesture.

It has been found that muscle recruitment during the squat can depend on joint position, ROM, and effort level (5, 11). Differences in the execution of the technical gesture of the squat can cause different muscle recruitment and joint moments (3). The scientific literature shows several studies that have quantified angular and linear kinematics during the execution of the BS (26–32). However, these studies have evaluated the biomechanics of different types of squats but, to the best of our knowledge, none of these have considered how these can vary at different load intensities.

This study aimed to evaluate the angular kinematics of the hip, knee, and ankle, and the linear kinematics of the barbell during the BS at different load intensities in powerlifters and weightlifters. As for the angular kinematics, we hypothesized a decrease in hip relative angle and in knee relative angle in the maximum flexion position as the load intensity increases while, as for the linear kinematics, we hypothesized a decrease in ascent speed and ascent acceleration as the load intensity increases, approaching the 1-RM.

## 2 Materials and methods

### 2.1 Study design

This is a cross-sectional study in which angular kinematics (of the hip, knee, and ankle) and linear kinematics (of the barbell) were measured during the execution of the BS at different load intensities (i.e., 60%, 70%, 80%, 90%, 100%) of the 1-RM of each participant previously estimated.

This study was in accordance with the principles of the Declaration of Helsinki for the use of people in research and was approved by the Bioethics Committee of the University of Palermo (n. 99/2022).

### 2.2 Participants

Seventeen powerlifters and weightlifters (*n* = 14 powerlifters; *n* = 3 weightlifters; m = 12, f = 5; age: 26.86 ± 8.93 years; weight: 73.29 ± 13.56 kg; height: 170.88 ± 8.15 cm) were enrolled from a sports club in Palermo, Italy.

To be eligible, participants had to meet the following inclusion criteria: (1) at least 3 years of continuous experience in powerlifting or weightlifting in regional or national competitions; (2) at least 3 workouts/week; (3) at least 1.5 of relative strength. Participants were not eligible if the following exclusion criteria were met: (1) minors; (2) musculoskeletal injuries in the 6 months prior to the study.

Participation in the study occurred voluntarily and after completing and signing an informed consent form.

### 2.3 Procedure

The procedure consisted of the following two phases: (1) the 1-RM of the BS of each participant was estimated in order to measure the maximum load intensity that each participant was able to lift; (2) 1-week after the administration of the 1-RM test, each participant was asked to perform 5 trials of the BS at different load intensities (i.e., 60%, 70%, 80%, 90%, 100%) of the 1-RM during which an action camera recorded the execution of each BS trial in the sagittal plane and, afterward, the videos were analyzed by measuring the range of motion (ROM) of hip, knee, and ankle for the angular kinematics, and the timing, speeds, and accelerations of the barbell for the linear kinematics. A rest period of at least 3 min between trials was set.

The procedure was carried out in the same place (the gym of the sports club), in the same time slot (from 2.00pm to 4.00pm), and by the same researcher.

### 2.4 1-RM test of the BS

The aim of the test was to estimate the 1-RM through a set in which each participant was able to perform a number of repetitions that fell between 5 and 10.

After a 10-min self-administered warm-up, each participant was asked the load with which was able to perform 10–12 repetitions. Then, each participant performed a set with a 20% load added and, if the number of repetitions was not within 5 and 10, a second set with an additional load of 20% was performed after a 5-min rest. Thus, the 1-RM was calculated using the Brzycki formula: Predicted 1-RM = weight lifted/[1.0278—(0.0278 * number of repetitions performed)] (33).

### 2.5 BS at different load intensities

First, each participant performed a self-selected warm-up, which was the usual warm-up they carry out before each competition. It lasted about 15 min and included joint mobility exercises, dynamic stretching, and back squats with no weight.

Hence, each participant performed one repetition of the low-bar BS for each load intensity in the following specific order: 60%, 70%, 80%, 90%, 100% of the 1-RM. A rest period of at least 3 min between trials was scheduled.

For the BS execution, the starting position was in standing position with the barbell positioned across the back resting on the shoulders. During the descent phase, each participant was asked to flex the hip, knee, and ankle joints until the crease of the hip was lower than the top of the knee. During the ascent phase, each participant was asked to extend the hip, knee, and ankle joints until reaching the standing position.

All 5 trials were performed using Olympic barbell and weight plates. All 5 trials were carried out without allowing equipment and by wearing technical shoes.

### 2.6 Kinematics of the BS

For the kinematic analysis of all 5 trials, an action camera (GoPro 9, GoPro Inc., San Mateo, CA, USA) was positioned laterally (to the left of the participants) recording the execution of each BS trial in the sagittal plane. In detail, the action camera, set at a capture rate of 60 fps (frames per second), was positioned horizontally with respect to the floor at a distance of 2 m using an appropriate support.

Five markers were placed in specific anatomical positions for each participant (acromion, greater trochanter, iliotibial hemirim, lateral malleolus, head of the 5th metatarsal) and one marker was placed at the left end of the barbell. Furthermore, for the subsequent calibration of the video analysis software, we marked a 100 cm horizontal line on the floor with tape.

The 5 recorded videos, one for each trial, were subsequently analyzed using Kinovea software which allowed the measurement of the angular and linear kinematics parameters at the different load intensities. The validity and reliability of the Kinovea software was previously studied (34–36).

### 2.7 Parameters of angular kinematics

The angular kinematics, manually performed, analyzed the relative value of angles generated between two segments (37). Angular values were calculated in the starting position (i.e., standing position), in the maximum depth position (i.e., full squat), and in the ending position of the BS (i.e., standing position). At the starting and ending of the BS execution the hip and knee angle were fully extended. The relative hip angle was measured considering the thigh and trunk segments, the relative knee angle was measured considering the thigh and leg segments, and the relative ankle angle was measured considering leg and foot segments. Both the hip and knee angle measurements were expressed as 180° at maximum hip and knee extension, respectively. The ankle angle measurements were expressed as 180° at maximum plantar flexion.

The parameters considered for the angular kinematics were the following: hip relative angle in the starting position, hip relative angle in the maximum flexion, hip relative angle in the ending position, knee relative angle in the starting position, knee relative angle in the maximum flexion, knee relative angle in the ending position, ankle relative angle in the starting position, ankle relative angle in the maximum flexion, ankle relative angle in the ending position.

### 2.8 Parameters of linear kinematics

The linear kinematics analyzes the values of timing, distance, speed, and acceleration (38). The entire BS execution was divided into a descent and an ascent phase and by evaluating the trajectory of the barbell it was possible to acquire timing, distance, speed, and acceleration of the barbell during the two phases (3). The descent phase began with the first observable downward movement of the barbell and ended at the lowest point reached. The ascent phase began with the first observable upward movement of the barbell and ended at the highest point reached.

The parameters considered for the linear kinematics were the following: total distance of descent, descent speed, vertical speed of descent, horizontal speed of descent, descent acceleration, vertical acceleration of descent, horizontal acceleration of descent, total distance of ascent, ascent speed, vertical speed of ascent, horizontal speed of ascent, ascent acceleration, vertical acceleration of ascent, horizontal acceleration of ascent.

### 2.9 Statistical analysis

The Shapiro–Wilk test was carried out to investigate the distribution of data. A descriptive analysis of angular and linear kinematics parameters was performed. Given the normal distribution of data, differences in angular and linear parameters among the BS trials at different load intensities (i.e., 60%, 70%, 80%, 90%, 100%) of the 1-RM were analyzed using the one-way repeated measures ANOVA. The Tukey's *post hoc* test was used to compute multiple comparisons between the BS trials. The effect size was calculated through the partial eta-squared. The statistical significance level was set at *p* < 0.05.

All statistical analyses were performed using Jamovi software package (version 2.3.28) (39).

## 3 Results

The descriptive analysis of the angular and linear kinematics parameters is reported in Tables 1, 2.

**Table 1:** Descriptive analysis of angular kinematics.

	60%	70%	80%	90%	100%
Hip relative angle SP (°)	161 ± 6.55	161 ± 8.87	160 ± 7.13	159 ± 6.67	159 ± 8.23
Hip relative angle MF (°)	50.8 ± 6.38	55.2 ± 7.44	54.1 ± 6.71	55.8 ± 6.70	56.1 ± 8.24
Hip relative angle EP (°)	161 ± 8.28	162 ± 10	161 ± 8.30	162 ± 7.63	161 ± 7.02
Knee relative angle SP (°)	168 ± 6.77	168 ± 7.49	167 ± 8.20	168 ± 7.02	167 ± 7.28
Knee relative angle MF (°)	50.7 ± 14.3	51.8 ± 11.4	55 ± 10.8	56.7 ± 11.5	55.3 ± 9.74
Knee relative angle EP (°)	169 ± 6.83	169 ± 6.23	169 ± 6.99	169 ± 6.76	169 ± 6.07
Ankle relative angle SP (°)	119 ± 4.21	121 ± 4.37	122 ± 5.42	120 ± 6.39	121 ± 5.70
Ankle relative angle MF (°)	83.5 ± 7.93	87.1 ± 6	87.1 ± 9.66	85.6 ± 8.11	86.2 ± 6.35
Ankle relative angle EP (°)	120 ± 6.06	122 ± 6.17	123 ± 6.73	119 ± 6.35	121 ± 4.84

SP, starting position; MF, maximum flexion; EP, ending position.

**Table 2:** Descriptive analysis of linear kinematics.

	60%	70%	80%	90%	100%
Descent speed (m/s)	0.909 ± 0.226	0.95 ± 0.272	0.991 ± 0.271	0.912 ± 0.257	0.922 ± 0.246
Vertical speed of descent (m/s)	−0.898 ± 0.229	−904 ± 0.266	−0.98 ± 0.267	−0.848 ± 0.418	−0.779 ± 0.549
Horizontal speed of descent (m/s)	−0.0753 ± 0.0558	−0.071 ± 0.076	−0.114 ± 0.142	−0.071 ± 0.039	−0.06 ± 0.074
Descent acceleration (m/s^2^)	−0.012 ± 0.192	0.038 ± 0.334	−0.007 ± 0.261	−0.139 ± 0.306	−0.137 ± 0.228
Vertical acceleration of descent (m/s^2^)	0.039 ± 0.165	−0.04 ± 0.362	0.03 ± 0.269	0.083 ± 0.342	0.173 ± 0.241
Horizontal acceleration of descent (m/s^2^)	0.036 ± 0.059	0.002 ± 0.08	0.018 ± 0.083	0.003 ± 0.062	0.015 ± 0.128
Total distance of descent (cm)	127 ± 11.7	127 ± 13.1	126 ± 15	122 ± 14.2	125 ± 12.9
Ascent speed (m/s)	1.20 ± 0.179	1.12 ± 0.216	1.02 ± 0.193	0.799 ± 0.182	0.616 ± 0.198
Vertical speed of ascent (m/s)	1.18 ± 0.181	1.07 ± 0.206	1.01 ± 0.189	0.789 ± 0.182	0.565 ± 0.232
Horizontal speed of ascent (m/s)	0.08 ± 0.072	0.08 ± 0.05	0.062 ± 0.06	0.041 ± 0.047	0.08 ± 0.181
Ascent acceleration (m/s^2^)	0.292 ± 0.427	0.375 ± 0.534	0.25 ± 0.432	0.212 ± 0.472	0.101 ± 0.166
Vertical acceleration of ascent (m/s^2^)	0.115 ± 0.653	0.311 ± 0.575	0.258 ± 0.422	0.160 ± 0.406	0.082 ± 0.185
Horizontal acceleration of ascent (m/s^2^)	0.096 ± 0.134	0.179 ± 0.155	0.156 ± 0.128	0.118 ± 0.105	0.042 ± 0.09
Total distance of ascent (cm)	130 ± 12.6	128 ± 12.8	129 ± 13.9	125 ± 12.8	131 ± 15.2

### 3.1 Angular kinematics

No significant differences were found in the angular kinematics parameters in the starting and ending positions among the 5 trials at different load intensities (Table 3). In the maximum flexion position, no significant differences were found in both the knee relative angle and the ankle relative angle parameters among the 5 trials at different load intensities, while, a significant difference was found in the hip relative angle [F_(4,52)_ = 3.01; *p* = 0.026; ƞ^2^partial = 0.188]. *Post hoc* multiple comparisons test revealed a significant increase from 60% to 70% (*p* = 0.007), from 60% to 90% (*p* = 0.011), and from 60% to 1-RM (*p* = 0.017), as reported in Table 4.

**Table 3:** Repeated measures ANOVA of angular kinematics.

	F_(4,52)_	p	ƞ^2^partial
Hip relative angle SP (°)	0.555	0.697	0.041
Hip relative angle MF (°)	**3**.**01**	**0**.**026**	**0**.**188**
Hip relative angle EP (°)	0.599	0.665	0.044
Knee relative angle SP (°)	1.16	0.340	0.082
Knee relative angle MF (°)	0.815	0.521	0.059
Knee relative angle EP (°)	0.206	0.934	0.016
Ankle relative angle SP (°)	1.67	1.171	0.114
Ankle relative angle MF (°)	1.43	0.239	0.099
Ankle relative angle EP (°)	1.83	0.138	0.123

SP, starting position; MF, maximum flexion; EP, ending position.

In bold the statistically significant values (*p* < 0.05).

**Table 4:** *Post hoc* multiple comparisons of the hip relative angle parameter in the maximum flexion.

Comparisons						
Hip relative angle (°)		Hip relative angle (°)	MD	SE	*t*	*p*
60%	vs	70%	−4.093	1.27	−3.2156	**0**.**007**
	vs	80%	−3.314	1.55	−2.1389	0.052
	vs	90%	−5.071	1.72	−2.9456	**0**.**011**
	vs	100%	−4.943	1.81	−2.7382	**0**.**017**
70%	vs	80%	0.779	1.60	0.4881	0.634
	vs	90%	−0.979	1.62	−0.6048	0.556
	vs	100%	−0.850	2.38	−0.3569	0.727
80%	vs	90%	−1.757	1.18	−1.4844	0.162
	vs	100%	−1.629	1.57	−1.0354	0.319
90%	vs	100%	0.129	1.89	0.0680	0.947

MD, mean difference; SE, standard error.

In bold the statistically significant values (*p* < 0.05).

### 3.2 Linear kinematics

In the descent phase, a significant difference was found in the parameter of the descent acceleration [F_(4,52)_ = 2.56; *p* = 0.049; ƞ^2^partial = 0.165], as reported in Table 5. *Post hoc* multiple comparisons test revealed a significant decrease from 70% to 1-RM (*p* = 0.028). In the ascent phase, a significant difference was found in the parameter of the ascent speed [F_(4,52)_ = 55.6; *p* < .001; ƞ^2^partial = 0.810] and vertical speed of ascent [F_(4,52)_ = 42.9; *p* < .001; ƞ^2^partial = 0.767]. As for the ascent speed, *post hoc* multiple comparisons test showed a significant decrease among the 5 trials at different load intensities except from 60% to 70% (Table 6). Regarding the vertical speed of ascent, *post hoc* multiple comparisons test detected a significant decrease from 60% to 80%, from 70% to 90%, from 70% to 1-RM (*p* < .001), from 80% to 90%, from 80% to 1-RM (*p* < .001), from 90% to 1-RM (*p* < .001).

**Table 5:** Repeated measures ANOVA of linear kinematics.

	F_(4,52)_	p	ƞ^2^partial
Descent speed (m/s)	1.15	0.345	0.081
Vertical speed of descent (m/s)	1.33	0.271	0.271
Horizontal speed of descent (m/s)	0.852	0.499	0.062
Descent acceleration (m/s^2^)	**2** **.** **56**	**0**.**049**	**0**.**165**
Vertical acceleration of descent (m/s^2^)	1.68	0.170	0.114
Horizontal acceleration of descent (m/s^2^)	0.838	0.507	0.061
Total distance of descent (cm)	1.14	0.350	0.080
Ascent speed (m/s)	**55**.**6**	**<**.**001**	**0**.**810**
Vertical speed of ascent (m/s)	**42**.**9**	**<**.**001**	**0**.**767**
Horizontal speed of ascent (m/s)	0.718	0.584	0.052
Ascent acceleration (m/s^2^)	1.10	0.366	0.078
Vertical acceleration of ascent (m/s^2^)	0.720	0.582	0.052
Horizontal acceleration of ascent (m/s^2^)	**4**.**89**	**0**.**002**	**0**.**273**
Total distance of ascent (cm)	1.56	0.199	0.107

In bold the statistically significant values ​​(*p* < 0.05).

**Table 6:** *Post hoc* multiple comparisons of the ascent speed parameter.

Comparisons						
Ascent speed (m/s)		Ascent speed (m/s)	MD	SE	*t*	*p*
60%	vs	70%	0.0628	0.0414	1.52	0.153
	vs	80%	0.1927	0.0309	6.24	**<** **.** **001**
	vs	90%	0.3816	0.0526	7.26	**<**.**001**
	vs	100%	0.6077	0.0646	9.41	**<**.**001**
70%	vs	80%	0.1299	0.0219	5.94	**<**.**001**
	vs	90%	0.3189	0.0502	6.35	**<**.**001**
	vs	100%	0.5449	0.0613	8.89	**<**.**001**
80%	vs	90%	0.1889	0.0423	4.47	**<**.**001**
	vs	100%	0.4150	0.0494	8.39	**<**.**001**
90%	vs	100%	0.2261	0.0393	5.75	**<**.**001**

MD, mean difference; SE, standard error.

In bold the statistically significant values ​​(*p* < 0.05).

## 4 Discussion

This study aimed to analyze angular and linear kinematics of the BS at different load intensities in powerlifting and weightlifting athletes. Our hypothesis was to detect a change in the execution technique of the BS as the percentage of load intensity increased, approaching the 1-RM. In detail, we hypothesized a decrease in hip relative angle and in knee relative angle in the maximum flexion position as the load intensity increases and a decrease in ascent speed and ascent acceleration as the load intensity increases. As detailed below, the hypothesis was partially confirmed as significant differences in angular and linear kinematics parameters were found.

### 4.1 Angular kinematics

Regarding the parameters of angular kinematics, our results showed no significant differences except for the hip relative angle in the maximum flexion position which decreased as load intensity increased. This result indicates that the participants, when performing the trials with load intensities close to the 1-RM, in the maximum flexion position tended to flex their hips less and to keep their thighs parallel to the ground (condition that allows the gesture to be considered valid during competitions). The reason why hip flexion was lower with loads close to 1-RM could be explained by a lower forward tilt of the trunk in the position of maximum flexion which, instead, occurred later (i.e., during the ascent phase). This interpretation is in line with the findings by Escamilla et al. who analyzed the ascent phase of the squat by dividing it into 3 parts (i.e., acceleration phase, sticking region, maximum strength) and showed that only in the second part (which represents the most difficult part of the lift in the ascent phase) athletes slightly increase the forward tilt trunk and hip flexion to increase the length of the hamstrings and other hip extensors in order to augment the ability of these muscles for generating force (3). The forward tilt of the trunk also allows a greater contribution from the back muscles. However, some studies have shown that experienced squatters can achieve better results and more favorable kinematics by keeping the trunk more upright (14, 29). Considering that the sample recruited for this study was composed of experienced athletes, our results are supported by these studies.

It should be noted that the differences in the angular kinematics of the gesture suggest that there can be variations in muscle recruitment. Indeed, the study by Da Silvia et al. investigating muscle activation in partial and full back squat showed a similar overall level of muscle activation of the quadriceps femoris, while a higher muscle activation of the gluteus maximum, biceps femoris, and erector spinae was found in partial vs. full back squat (40). Participants in our study reached the full squat only at lower load intensities and probably did not bring their thighs beyond parallel at higher load intensities to have greater involvement of the abovementioned muscles. In this regard, many studies in the literature have shown that muscle activity progressively increases as the knees flex and decreases as the knees extend (5).

No significant differences were found between the angular kinematics parameters in the starting and ending position of the BS among the 5 trials at different load intensities because the hip and knee were almost fully extended, and this is not influenced by the load intensity.

### 4.2 Linear kinematics

Regarding the descent phase, no significant differences were detected in the parameters of linear kinematics except for the descent acceleration. This means that this phase of the squat is not influenced by the load intensity probably because the entire descent phase is carried out in favor of gravity, in the opposite way to the ascent phase in which is required to counteract the force of gravity.

Regarding the ascent phase, we found a significant difference in the ascent speed which decreases significantly approaching the 1-RM. This result is in line with the study by Trybulski et al. which aimed to determine the duration and velocity of the movement that occurs during the volitional movement tempo of squat and bench press showing that the increase in load caused a decrease in the bar velocity (41). Bentley et al. analyzed the influence of different lifting cadences (i.e., fast cadence, medium cadence, slow cadence) on ground reaction forces during the squat (42). The authors showed that the range of ground reaction force is greater for squats with greater ascent speed. Thus, it would appear that athletes who are slower in the ascent phase of the squat have lower ground reaction force. A previous study explored the influence of two different cadences (i.e., 1 s and 2 s) on tibiofemoral joint force during a half squat reporting that squat cadence influences fatigue in the medio-lateral shear and compressive forces of the knee joint (43). There was a 25% increase in the medio-lateral shear for the slow cadence and a slight decrease for the fast cadence. The compressive forces showed a 52% increase for the slow cadence and a 22% increase for the fast cadence. Therefore, trials at higher intensities load performed at lower cadence can increase tibiofemoral shear forces which can induce cruciate ligaments injuries, while excessive tibiofemoral compressive forces can be deleterious to the menisci and articular cartilage (5). Furthermore, in our recent study we investigated the postural control of powerlifters and weightlifters who performed the BS at different intensities (25). The results of this study are in line with the abovementioned studies since, as the load increased, a worsening of postural control was detected, increasing the risk of injury.

## 5 Conclusion

Our results underline that squat performed at different load intensities shows changes in both the angular and linear kinematics and, consequently, in the execution of the technical gesture influencing the related muscles recruited. These results can be useful for athletes, coaches, and trainers involved in strength and power sports in which this exercise is included in competitions (e.g., in powerlifting) and in training (e.g., in weightlifting) emphasizing the importance of performing the kinematic analysis of the exercises in sports. These results are also of interest to sports medical doctors and physiotherapists who propose, among others, closed kinetic chain exercises, such as the squat, for knee rehabilitation (5). In fact, considering the complexity of the squat, understanding the biomechanics of the squat is of great importance both to achieve the maximum performance and to reduce the risk of injury (16, 44). The main strength of this study is that, to the best of our knowledge, it is the first in the literature to analyze how the angular and linear kinematics of the BS change at different load intensities. Among the limitations, it should be mentioned the small sample size and the fact that the kinematic analysis performed is two-dimensional.

## Data Availability

The raw data supporting the conclusions of this article will be made available by the authors, without undue reservation.
